# Novel Magnetic Resonance Imaging Tools for Hypertrophic Cardiomyopathy Risk Stratification

**DOI:** 10.3390/life14020200

**Published:** 2024-01-30

**Authors:** Fahad Alajmi, Mehima Kang, James Dundas, Alexander Haenel, Jeremy Parker, Philipp Blanke, Fionn Coghlan, John King Khoo, Abdulaziz A. Bin Zaid, Amrit Singh, Bobby Heydari, Darwin Yeung, Thomas M. Roston, Kevin Ong, Jonathon Leipsic, Zachary Laksman

**Affiliations:** 1 Center for Cardiovascular Innovation, Division of Cardiology, Department of Medicine, University of British Columbia, 2775 Laurel St, 9th Floor, Vancouver, BC V5Z 1M9, Canada; mehima.kang@gmail.com (M.K.); ahaenel@providencehealth.bc.ca (A.H.); jeremy.parker@ubc.ca (J.P.); phil.blanke@gmail.com (P.B.); coughlan@providencehealth.bc.ca (F.C.); jkhoo1@providencehealth.bc.ca (J.K.K.); dr.abdulazizbinzaid@gmail.com (A.A.B.Z.); bobby.heydari@ucalgary.ca (B.H.); darwin.yeung@ubc.ca (D.Y.); rostontm@alumni.ubc.ca (T.M.R.); kong3@providencehealth.bc.ca (K.O.); 2 Department of Radiology, University of British Columbia, 2775 Laurel Street, 11th Floor, Vancouver, BC V5Z 1M9, Canada; james.dundas@nhs.net (J.D.); jonathon.leipsic@ubc.ca (J.L.); 3 Department of Cardiology, North Tees and Hartlepool NHS Foundation Trust, Hardwick Rd, Hardwick, Stockton-on-Tees TS19 8PE, UK; 4 Department of Anesthesiology, Pharmacology and Therapeutics, The University of British Columbia, Medical Sciences, 2176 Health Sciences Mall Block C217, Vancouver, BC V6T 2A1, Canada; amrit.singh@hli.ubc.ca

**Keywords:** hypertrophic cardiomyopathy, cardiac magnetic resonance imaging, prognosis, T1, T2, feature tracking

## Abstract

Hypertrophic cardiomyopathy (HCM) is a common genetic disorder with a well described risk of sudden cardiac death; however, risk stratification has remained a challenge. Recently, novel parameters in cardiac magnetic resonance imaging (CMR) have shown promise in helping to improve upon current risk stratification paradigms. In this manuscript, we have reviewed novel CMR risk markers and their utility in HCM. The results of the review showed that T1, extracellular volume, CMR feature tracking, and other miscellaneous novel CMR variables have the potential to improve sudden death risk stratification and may have additional roles in diagnosis and prognosis. The strengths and weaknesses of these imaging techniques, and their potential utility and implementation in HCM risk stratification are discussed.

## 1. Introduction

Hypertrophic cardiomyopathy (HCM) is a common genetic disorder characterized by increased thickness of the left ventricular wall, not attributable to increased afterload [1]. Sudden cardiac death (SCD) is a feared complication of HCM, as outlined in the European Society of Cardiology (ESC) 2022 and 2023 guidelines, which describe an annual mortality rate of 1% to 2% and an annual rate of SCD or appropriate implantable cardioverter defibrillator therapy of 0.8% [2,3]. SCD is defined as sudden and unexpected death, presumed due to either cardiac arrythmia or hemodynamic collapse [4], occurring either within an hour of symptom onset, or being found dead within 24 h of an asymptomatic period. Known risk factors for SCD in HCM, as proposed by the American Heart Association/American College of Cardiology (AHA/ACC) and outlined in Table 1, include a family history of sudden cardiac death, left ventricular hypertrophy ≥30 mm, and extensive late gadolinium enhancement ≥15% of left ventricular mass [5]. In current clinical practice, these risk factors are often combined using risk prediction calculators [6] to aid decision- making regarding interventions to reduce SCD risk, such as implantation of an implantable cardioverter–defibrillator (ICD). However, these parameters fail to include a certain subset of HCM patients who experience SCD, while overestimating the risk in others, and may not outperform existing CMR criteria, such as extensive LGE alone [7]. To fill the knowledge gaps and thus improve risk stratification, the field has increasingly relied on cardiac genetics and CMR. A number of recently introduced CMR-based techniques have shown promise to improve risk prediction but have not yet been included in current guidelines or position statements. There is, however, a pressing clinical need for refinement in risk stratification strategies to effectively guide the implantation of ICDs, with the ultimate goal of optimizing the balance between sensitivity and specificity [8]. We sought to review the recent literature describing these novel CMR parameters, their potential role in improving sudden death risk stratification, and their potential applications in the diagnosis and prognosis of patients with suspected HCM.

## 2. Materials and Methods

A literature review was conducted according to the Preferred Reporting Items for Systematic Reviews and Meta-Analyses (PRISMA) scoping review guidelines for CMR-based assessment of risk stratification in HCM. The timeframe of the articles reviewed was between January 2019 and October 2022, as the period preceding this was previously reviewed in depth and presented in the 2020 American Heart Association HCM guidelines [5]. The search keywords “Cardiomyopathy, Hypertrophic” AND “Magnetic Resonance Imaging” were used to query the PubMed database. The inclusion and exclusion criteria are outlined in Table 2 below.

## 3. Results

**Figure 1:**
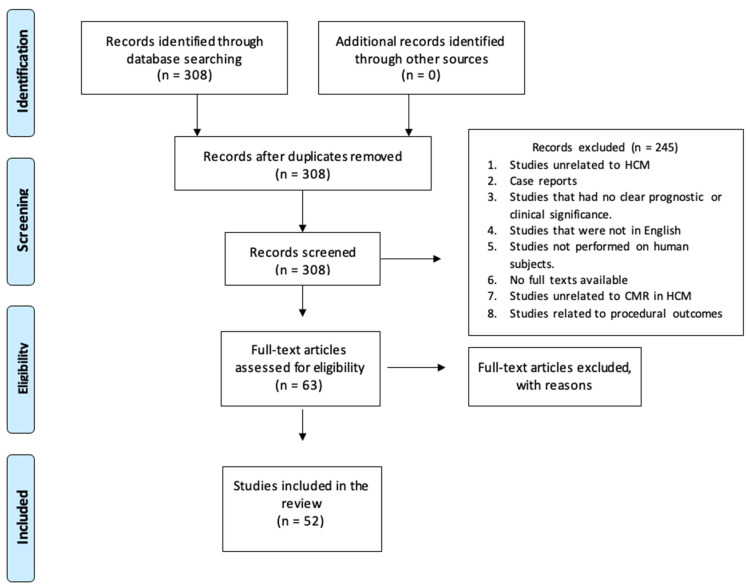
A flow diagram for review methodology.

Initial PubMed results from these search terms came to 308 studies. An abstract review was performed by a single independent reviewer using the systematic review methodology, and application of the inclusion and exclusion criteria reduced this to 63 studies. Further results were excluded for being related to surgical and procedural outcomes, those focused specifically on other factors such as ECG findings and not CMR, and those with no full text available. The final count of relevant articles that met all inclusion criteria was 52 (Figure 1). These studies were reviewed for correlations between our line of inquiry and the available data.

Our initial analysis of the articles deemed relevant by the literature review guidelines produced three main categories: T1 and extracellular volume (ECV), T2 and myocardial edema, and CMR strain methods that were predominantly related to feature tracking. A number of studies did not fit any of these criteria and were distinct from each other and were therefore grouped together as Other Parameters.

### 3.1. T1 Mapping and Extracellular Volume

Longitudinal T1 relaxation times are an intrinsic property of biological tissues in a magnetic field and describe the time required for protons within tissues to recover back into alignment with the static B_0_ field of the MRI scanner following excitation with a radiofrequency energy pulse. Different tissues (e.g., fat, myocardium, blood) have different inherent T1 relaxation times, and these are further modified by administration of gadolinium-based contrast agents or the presence of disease states, such as the development of fibrosis within the myocardium. Measurement of true myocardial T1 relaxation curves is impractically time-consuming; however, they can be estimated using multiple available sequences (MOLLI, shortened MOLLI, SASHA, SAPPHIRE) with reasonable accuracy. T1 mapping denotes the estimation of pre-contrast (native) T1 times at the individual pixel level, allowing quantitative assessment of diffuse pathology (e.g., interstitial fibrosis) without requiring contrast administration. T1 mapping of both blood pool (correcting for hematocrit) and myocardium before and after administration of gadolinium contrast allows estimation of the myocardial extracellular volume (ECV) fraction [9]. Disease states such as extensive fibrosis, and infiltrative pathologies such as cardiac amyloidosis particularly expand the extracellular space, and so increase ECV.

Multiple studies have highlighted that higher T1 and ECV values in the HCM population compared to a control group were correlated with myocardial fibrosis, refs. [10,11,12,13,14,15] suggesting these parameters are useful diagnostically to help differentiate HCM from other causes of LVH, such as athletic remodeling, where minimal myocardial fibrosis is expected.

**Figure 2:**
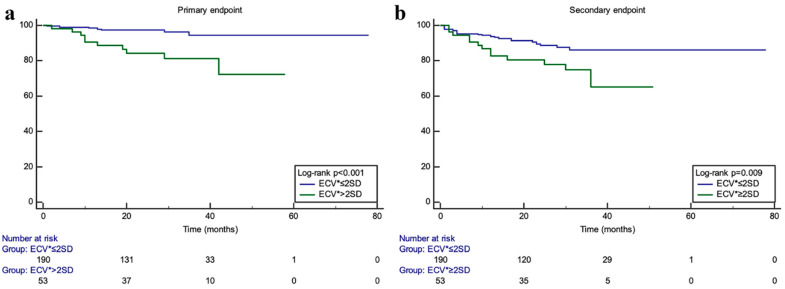
Kaplan–Meier curves from Li et al. [16] demonstrating survival free from events for the primary endpoint (composite of CV death, heart transplant, aborted SCD, and syncope requiring CPR) in KM curve (**a**), and for the secondary endpoint (heart failure hospitalizations) in KM curve (**b**), for subjects with ECV > 2 SD above mean (green curve) vs. those with ECV ≤ 2SD above mean (blue curve) (reproduced with permission).

Li et al. [16] demonstrated that HCM patients with an elevated ECV had a significant increase in primary cardiovascular endpoints (cardiac death, heart transplant, aborted sudden death, and cardiopulmonary resuscitation after syncope) and secondary cardiovascular endpoint (heart failure hospitalization) with a *p* value < 0.01 for primary outcomes and =0.009 for secondary outcomes (Figure 2).

Xu et al. [17] demonstrated elevated T1 and ECV values in non-obstructive HCM compared to healthy controls, even in the absence of LGE, and found a strong association between these parameters and increased LV mass index. They also compared these parameters to outcomes and found an association between elevated T1 or ECV and SCD in a univariable analysis, although there were few events during follow-up (5 SCD/258 patients, 1.9%). These findings support the use of both T1 and ECV mapping for both diagnostic and SCD prediction purposes and, in particular, suggest additive utility alongside existing LGE sequences, given that they studied patients who would ordinarily be classified as low-risk, given the absence of both outflow obstruction and LGE. A meta-analysis of this topic conducted by Raiker et al. [18] concluded that ECV ≥ 34% was a more powerful predictor of SCD and NSVT (non-sustained ventricular tachycardia) and was more effective in identifying HCM patients with NSVT or syncope compared to LGE and post-contrast T1.

Wang et al. [10] compared a novel non-contrast T1ρ (T1-rho) dispersion map technique called myocardial fibrosis index (mFI) to post-contrast ECV mapping for the diagnosis of diffuse fibrosis in individuals with HCM. The ability of the T1ρ dispersion mFI to differentiate fibrosis content in both normal-thickness hypertrophic cardiomyopathy (HCM-N) (defined as a maximal end diastolic wall thickness of <15 mm) and hypertrophied hypertrophic cardiomyopathy (HCM-H) was either equal to or notably improved compared to the ECV, as evident from their receiver operating characteristic curves. This study therefore concluded that, since no contrast is used, patients with renal insufficiency may benefit from CMR T1ρ dispersion mFI to identify diffuse fibrosis.

### 3.2. T2-Weighted CMR Imaging and T2 Mapping

T2 is another intrinsic property of tissue in a magnetic field and represents the decay of lateral magnetization (as opposed to longitudinal magnetization in T1). T2 decay is prolonged in tissues with increased water content, so T2-weighted imaging sequences (e.g., short-tau inversion recovery, STIR) have long been used for the qualitative assessment of myocardial edema. Similar to T1 mapping, T2 mapping sequences are now also used for quantitative edema evaluation [19]. While myocardial edema is not specific to HCM and is traditionally associated with acute pathologies such as acute myocardial infarction or myocarditis, there has been recent interest in the utility of T2-weighted imaging in chronic cardiomyopathies such as HCM.

**Figure 3:**
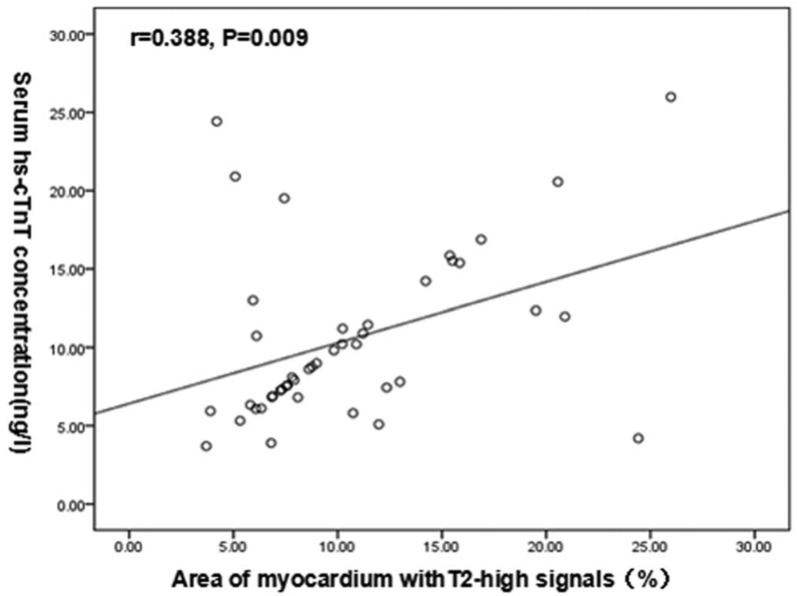
Scatter plot from Chen et al. [20] demonstrating the association between the percentage of myocardium with a T2 signal and the degree of hs-cTnT level elevation. (Reproduced with permission). Medicine (Baltimore). 2020 Jun 5; 99(23): e20134. Published online 2020 Jun 5. doi: 10.1097/MD.0000000000020134. Copyright © 2020 the Author(s). Published by Wolters Kluwer Health, Inc. Creative Commons Attribution License 4.0 (CCBY).

Chen et al. [20] investigated the relationship between T2 signal on CMR and high-sensitivity cardiac troponin T (hs-cTnT), demonstrating a strong association between increasing hs-cTnT levels and both the number of cardiac segments with elevated T2 (*p* = 0.002) and the percent of myocardium involved (Pearson correlation: r = 0.388, *p* = 0.009, Figure 3). They also noted that segments with elevated T2 were significantly more hypertrophied than those without, suggesting the possibility that edema may be a marker of active disease in HCM. Logistic regression analysis identified the percentage of myocardium with a high T2 signal to be the only independent predictor of elevated hs-cTnT (OR: 0.707, 95%CI: 0.505–0.981, *p* = 0.038). These findings suggest that prolonged T2 decay is an indicator of myocardial damage in hypertrophic cardiomyopathy and merits further assessment as a biomarker in this condition.

Similarly, Cramer et al. [21] identified an association between post-exercise troponin elevation and high T2 signals in hypertrophic cardiomyopathy patients. They described elevated T2 signal as the only independent predictor of troponin rise (odds ratio 7.9; 95%CI 2.7–23.3; *p* < 0.001), thereby concluding that T2-weighted imaging can recognize cohorts of vulnerable patients with active disease who may be at risk during exercise. This could be of particular use given the paucity of evidence [22,23] supporting common recommendations to reduce or avoid exercise in HCM due to perceived SCD risk.

### 3.3. CMR Feature Tracking and Other Strain Methods

Feature tracking (FT) is an emerging tissue-tracking technique using post-processing of CMR cine sequences already acquired for ventricular morphology and function. Similar to the now widely-used speckle-tracking strain analysis in echocardiography, this technique involves quantitative evaluation of myocardial deformation, generating indices such as strain and strain rate for cardiac longitudinal, radial, and torsional (circumferential) motion. Given the good spatial resolution of balanced steady-state free precession (bSSFP) cine imaging with whole-heart coverage, these indices can be generated for individual LV myocardial layers (e.g., epicardial vs. endocardial strain) as well as applied to the thin-walled atria and right ventricle. Strain can be calculated in 2D (with reference to fixed LV geometry) or in 3D, with the latter potentially superior in complex and unusual anatomy. The concept that strain analysis might provide additional information beyond traditional global and segmental functional analysis is widely acknowledged in the current literature [24].

Xu et al. report that CMR-FT can be used to recognize myocardial dysfunction in HCM patients even with normal LV wall thickness and preserved LVEF [24]. Furthermore, they propose that the differences in epicardial and endocardial global circumferential strain can reflect HCM disease status, including both preclinical and overt. Xu et al. found that impaired left ventricular strain in HCM patients could be correlated with poor cardiac outcomes in terms of cardiovascular mortality and HF.

Song et al. included 123 obstructive HCM patients who underwent CMR prior to surgical myectomy, investigating the relationship between CMR-FT strain parameters and histopathologic myocardial fibrosis post-myectomy [25]. They found that circumferential (*p* = 0.003), longitudinal (*p* = 0.001), and radial (*p* = 0.02) strain in the interventricular septum were all significantly reduced in HCM patients with confirmed histological myocardial fibrosis despite a lack of LGE compared to those without fibrosis. Following multivariate analysis, septal longitudinal strain was independently associated with both fibrosis (*β* coefficient 0.19, 95%CI 0.05–0.34, *p =* 0.01) and ventricular arrhythmias (OR 1.10, 95%CI 1.01–1.19, *p* = 0.02), which was even incremental to LGE for both outcomes. This additive risk prediction may be of particular interest, given that the technique does not require further image acquisition over standard CMR protocols.

Cavus et al. retrospectively studied 144 HCM patients and 16 healthy controls and again found that multiple left ventricular strain parameters (LV long-axis longitudinal and LV short-axis radial) and, additionally, left atrial longitudinal strain, were all impaired in HCM patients compared to controls despite normal LV ejection fraction [26]. They also compared this to serum cardiac biomarkers and found an association between impaired LA and LV strain and elevated NT-proBNP and hsTnT in HCM when compared to HCM patients with normal biomarker levels. Only a modest association was observed between LV and LA cardiac magnetic resonance feature tracking (CMR-FT) values and the levels of NT-proBNP and hsTnT, and right ventricular strain was not impaired in HCM subjects compared to controls.

To further examine atrial strain, Zhou et al. investigated the interaction of CMR-FT LA strain and clinical outcomes in 60 HCM patients and 60 hypertension patients with normal LA size [27]. LA strain was more sensitive than LV longitudinal strain for evaluation of their primary endpoint, a composite of all-cause death, stroke, new-onset or worsening heart failure leading to hospitalization, and paroxysmal or persistent atrial fibrillation. Furthermore, their Cox regression analyses found that impaired LA reservoir and booster pump strain were associated with clinical outcomes in patients at the early stage of hypertension (HTN) and HCM (*p* < 0.05).

Heart failure with preserved ejection fraction (HFpEF) is common in HCM and is associated with adverse outcomes, including all-cause mortality [28]. Shi et al. used CMR-FT to determine the association between HFpEF and left atrial function in HCM patients. Left atrial phasic strain was able to differentiate between HCM patients with heart failure with preserved ejection fraction (HFpEF) and those without and could further categorize the severity of patients with HFpEF, whereas, in their population, LV global longitudinal strain could not [29]. LA reservoir (*β* = 0.90 [0.85–0.96]), conduit (*β* = 0.93 [0.87–0.99]), and booster (*β* = 0.86 [0.78–0.95]) strain were all independently associated with HFpEF. They concluded that the phasic function of the left atrium (LA) was notably compromised in patients with HCM and HFpEF, lending further credence to the concept of atrial indices providing additive value beyond simple LA size and ventricular function.

Yang et al. evaluated a novel automated analysis tool for LA strain (designated fast left atrial long-axis strain, LA-LAS) [30]. This technique tracks the distance between the midpoint of the posterior left atrial wall and the left atrioventricular junction, requiring only three anatomical points to be designated, rather than up to 48 points in standard LA CMR-FT. A total of 359 HCM patients and 100 healthy controls participated, and standard LA strain parameters from the fast LA-LAS parameter were compared to a MACE composite of CV death, resuscitated cardiac arrest, SCD aborted by ICD discharge, and heart failure hospitalization. They found that both fast LA reservoir strain and fast LA conduit strain better predicted the composite outcome compared to LA size, LA volume index, and even the presence of LV LGE. They also reported that fast LA strain appears highly reproducible (ICC: 0.95−0.96; COV: 7.2–9.6%).

Finally, Barbosa et al. investigated the relationship between LV 2D global strain measurements using CMR-FT, morphological characteristics, and HCM prognostic markers [31]. They concluded that a more severe HCM phenotype, the presence of ventricular arrythmias, and a higher predicted risk of SCD were all linked to impaired CMR-FT strain measures.

### 3.4. Other CMR Parameters

Mahmod et al. undertook CMR in 290 HCM patients with LVEF ≥ 55% and 30 age- and sex-matched controls, with clinical follow-up for a median of 4.4 years and a repeat CMR in a sub-group of 63 patients [32]. They found that RV longitudinal strain was an independent predictor of non-sustained ventricular tachycardia (NSVT) [HR 1.05 (95% CI 1.01–1.09), *p* = 0.029] and that right ventricular ejection fraction was a predictor for non-sustained ventricular tachycardia and other cardiovascular events, including heart failure outcome and cardiovascular death. An association between global longitudinal strain and NSVT was also demonstrated.

Abnormalities in myocardial trabeculation, including hypertrabeculation [33] and multiple myocardial crypts [34], are well-described in hypertrophic cardiomyopathy, although their significance remains unclear. Wang et al. investigated the prognostic significance of myocardial trabecular complexity using fractal analysis in 378 individuals with HCM and 100 age- and gender-matched healthy controls [35]. Standard bSSFP cine sequences were post-processed to quantitatively estimate the fractal dimension (FD), a unitless measure of trabecular complexity that ranges from 1 to 2. They found that increased LV maximal apical FD ≥ 1.325 was associated with both the primary endpoint (composite of all-cause mortality and aborted SCD) and the secondary endpoint of heart failure hospitalization in participants with HCM.

Excess epicardial adipose tissue (EAT) has been associated with atrial fibrillation (AF) in the general population [36], and Zhou et al. used CMR to evaluate this association in HCM. Following multivariable regression, they found that increased EAT index, LA volume index, and LVEF were all independent predictors of AF, and that integration of all three parameters demonstrated high discriminatory performance (sensitivity 94.4%, specificity of 69.3%, AUC = 0.864, 95%CI 0.771–0.958) [37]. However, as they did not seek to evaluate associations with other adverse outcomes, the utility of this parameter in SCD risk stratification remains unknown.

There have been studies assessing the role of stress CMR in cardiovascular outcomes, especially in patients with coronary artery disease (CAD). Antiochos et al. (2022) [38] conducted a retrospective study titled “Prognostic Value of Stress Cardiac Magnetic Resonance in Patients With Known Coronary Artery Disease” with the aim of determining whether stress cardiac magnetic resonance (CMR) can provide clinically relevant risk reclassification for patients with established coronary artery disease (CAD) in a multicenter setting in the United States. Even though the majority of stress CMR studies have been assessing for CAD, there have been studies assessing the role of stress CMR in microvascular perfusion abnormalities in the HCM population. In a study performed by Kim et al. (2020) [39] titled “Prevalence and clinical significance of cardiovascular magnetic resonance adenosine stress-induced myocardial perfusion defect in hypertrophic cardiomyopathy”, among individuals diagnosed with hypertrophic cardiomyopathy (HCM), over 40% exhibited adenosine stress perfusion defects on cardiac magnetic resonance (CMR). There was an association between perfusion defect noted and with non-sustained ventricular tachycardia (NSVT), increased left ventricular (LV) mass index, and the presence of apical aneurysms.

## 4. Discussion

The studies highlighted have shown that recently introduced CMR techniques have the potential to provide improved prognostic information for patients with HCM. T1 and extracellular volume have been shown to be early markers of myocardial fibrosis in HCM patients and to have an association with adverse outcomes. Importantly, both T1 and ECV predicted MACE, but also specifically SCD, even in patients without LGE. This implies that we may have a spectrum of parameters that enable better risk stratification early in the disease prior to the development of overt LGE and that are applicable both with and without contrast administration. The latter may be advantageous in resource-limited settings, where both gadolinium contrast agent cost and the additional scanner time are at a premium, as well as in patients with renal impairment or contrast allergy. These findings are in line with the experience with T1 and ECV in multiple other disease states [40], such as dilated cardiomyopathy [41] and cardiac amyloidosis, where they are becoming routinely used in clinical practice.

Abnormalities in T2 in HCM were associated with serum biomarkers of myocardial injury. These studies are of interest, particularly because they may suggest a more active disease process than has been traditionally postulated in HCM. Theoretically, this may represent a therapeutic target for novel agents, but it might also identify patients who could benefit from measures to reduce SCD (e.g., exercise restriction during periods of active disease/myocardial injury) without exposing the wider HCM population to the downsides of these interventions. However, given that the studies included here only compared myocardial T2 to serum troponin values, it remains unclear whether T2 can provide additive information over troponin measurement alone, especially given the extremely high sensitivity of modern troponin assays as well as their lower cost and greater availability compared to CMR.

Regarding strain measurements from CMR feature tracking, not only were there associations with histological fibrosis and increased risk of ventricular arrythmias, but there were also significant findings of early atrial and ventricular dysfunction prior to the development of LGE or reduced ejection fraction. Some studies also validated simplified, easier-to-implement strain techniques, such as three-point fast LA long-axis strain, which may help overcome the downside of strain parameters that require more complex and time-consuming post-processing.

While some of the studies included did not specifically address the primary question of SCD risk, they may still be able to contribute to decision-making for HCM patients. For example, development of either HFpEF or atrial fibrillation is associated with worse outcomes but not with SCD; therefore, if left atrial strain and epicardial adipose tissue parameters can predict these complications, they could further inform patient and clinician decision-making. Small to moderate apical aneurysms, especially those with a thin wall, have been underdiagnosed and missed with the use of echocardiography. CMR has provided an advantage in the detection and diagnosis of these apical aneurysms [42]. In the era of novel HCM therapeutics such as mavacamten and future potential disease-modifying drugs, these imaging biomarkers may be used for patient selection or monitoring for response, so more data correlating their relationship with patient-centered clinical outcomes will be useful.

Of key importance, all of the main technique groups are relatively easily translated into modern CMR practice. The CMR-FT, fractal analysis, and EAT tools are all post-processed from standard workhorse cine sequences used for volumetric assessment of LV function. In line with other facets of CMR interpretation, some of these analyses are increasingly simplified and partially or fully automated with AI assistance using commercially available software. If only a single septal segment is to be analyzed, T1, ECV, and T2 mapping images can be acquired in three short breath-holds (one breath-held acquisition for each), adding minimal scan time to a standard CMR protocol. For a more comprehensive assessment, 16 AHA-segment coverage is feasible in nine breath-holds (three short-axis slices each at the base, mid-chamber, and apex).

While image acquisition is entirely feasible, the post-processing and reporting will be time-consuming where numerous parameters are being calculated, so understanding their relative value is likely to be crucial for widespread adoption. Additionally, the value of individual measures may vary at different stages of disease; for example, measures that are able to predict risk prior to the development of overt LGE may lose value later in the disease process when there is a high burden of LGE or significant systolic impairment. Building and validating a multi-modality risk prediction model is a key research question and one that could benefit from a machine-learning approach. In light of the potential future implications of this study’s results, we aim to explore their applicability in specialized patient populations, such as post-myectomy patients or patients that have been treated with mavacamten, as well as assess trends in CMR measures through repeated scans, offering avenues for further research and questions to be addressed. Of note, not all of these studies will be applicable, as some of these centers use the 3.0 T MRI machine and others are still using the 1.5 T.

The implementation of serial cardiac magnetic resonance (CMR) assessments over time serves as a pivotal aspect in research endeavors. For future studies, centers can establish a baseline using the aforementioned novel parameters in this paper and the appropriate monitoring spanning from six months up to five years to assess changes related to prognosis and outcomes. This longitudinal approach is integral to any research project, as it not only provides a foundational understanding but also forms the basis for replicable investigations. A commitment to serial assessments ensures a nuanced exploration of the prognostic and diagnostic potential inherent in long-term CMR monitoring, contributing significantly to the advancement of cardiovascular research.

## 5. Limitations

Given the broad nature of scoping reviews, the included studies vary widely in terms of methodologies, populations, and outcomes assessed. This heterogeneity makes meta-analysis (beyond a narrative synthesis such as our review) challenging.

Of note, several studies used composite outcomes, such as MACE. While this is common practice in cardiovascular trials, not all MACE components are amenable to interventions designed to reduce SCD, such as heart failure hospitalizations or non-sudden cardiovascular deaths likely attributable to pump failure or coronary artery disease. This limits the applicability of these studies when developing an SCD risk prediction model or calculator.

Furthermore, some study conclusions were based on a limited sample size, from single-center studies, and would benefit from further validation in larger multi-center datasets. For many of the mentioned studies, the demographic ethnicity of the population was not provided, again potentially limiting its applicability to other populations.

Several of the techniques were evaluated using novel or proprietary software, and it is unknown whether conclusions remain valid using software or MRI sequences from other vendors and whether the same normal ranges are applicable. For example, some echocardiographic strain indices are known to vary between vendors [43], so this could also be true of CMR feature tracking.

Similarly, native T1 normal values are known to vary not just by magnetic field strength but are unique to individual MRI scanners, thus compounding the limitation of single-center studies performed on a single magnet. To become a meaningful parameter in clinical practice for HCM risk stratification, standardization in quantification and reporting of native myocardial T1 values across different CMR hardware/software combinations and field strengths would be preferable [44].

## 6. Conclusions

In conclusion, there is growing evidence to support the inclusion of novel CMR parameters in the clinical risk stratification of sudden death in HCM patients. Further refinement of risk prediction using these parameters has the potential to reduce morbidity and mortality and drive new research into prognostication as well as the monitoring of disease-modifying drugs. In particular, with increasing research and experience, T1, extracellular volume, T2, and CMR feature tracking have the potential to influence future guidelines and clinical care pathways.

## Figures and Tables

**Table 1:** Demonstrates known risk factors for SCD in HCM, as proposed by the American Heart Association/American College of Cardiology (AHA/ACC) [5].

Risk Factors for Sudden Cardiac Death (SCD) in Hypertrophic Cardiomyopathy
1. Family history of sudden death in HCM
2. Massive left ventricular hypertrophy (LVH) ≥ 30 mm
3. Unexplained syncope
4. HCM with left ventricular dysfunction < 50%
5. Presence of left ventricular apical aneurysm
6. Extensive late gadolinium enhancement (LGE) on CMR imaging (≥15% of left ventricular mass)
7. Non sustained ventricular tachycardia (VT) on ambulatory monitoring

**Table 2:** Inclusion and exclusion criteria used during our systematic review.

Inclusion Criteria:	Exclusion Criteria:
Studies published in English	Studies unrelated to HCM
Studies that looked at the association of novel CMR tools that were not employed in the latest American guidelines	Studies that had no clear prognostic or clinical significance in HCM patients
Studies that looked at the association of novel CMR tools with cardiovascular outcomes and prognosis, cardiac remodeling, hemodynamics, fibrosis and cardiac biomarkers	Case reports
	Studies that were not published in English
	Studies that were not performed in human subjects
	No full texts available
	Studies unrelated to CMR in HCM
	Studies related to procedural outcomes

## Data Availability

The original contributions presented in the study are included in the article, further inquiries can be directed to the corresponding authors.

## Abbreviations

HCM	Hypertrophic cardiomyopathy
ESC	European Society of Cardiology
SCD	Sudden cardiac death
LVH	Massive left ventricular hypertrophy
LGE	Late gadolinium enhancement
CMR	Cardiac magnetic resonance
VT	Ventricular tachycardia
ECV	Extracellular volume
NSVT	Non-sustained ventricular tachycardia
CMR-FT	Cardiac magnetic resonance feature tracking
LVEF	Left ventricular ejection fraction
HFpEF	Heart failure with preserved ejection fraction
hsTnT	High-sensitivity troponin T

## References

[1] Makavos G., Κairis C., Tselegkidi M.E., Karamitsos T., Rigopoulos A.G., Noutsias M., Ikonomidis I. (2019). Hypertrophic cardiomyopathy: An updated review on diagnosis, prognosis, and treatment. Heart Fail. Rev..

[2] Arbelo E., Protonotarios A., Gimeno J.R., Arbustini E., Barriales-Villa R., Basso C., Bezzina C.R., Biagini E., Blom N.A., de Boer R.A. (2023). 2023 ESC Guidelines for the management of cardiomyopathies. Eur. Heart J..

[3] Zeppenfeld K., Tfelt-Hansen J., de Riva M., Winkel B.G., Behr E.R., Blom N.A., Charron P., Corrado D., Dagres N., de Chillou C. (2022). 2022 ESC Guidelines for the management of patients with ventricular arrhythmias and the prevention of sudden cardiac death. Eur. Heart J..

[4] Jazayeri M.A., Emert M.P. (2019). Sudden Cardiac Death: Who Is at Risk?. Med. Clin. N. Am..

[5] Ommen S.R., Mital S., Burke M.A., Day S.M., Deswal A., Elliott P., Evanovich L.L., Hung J., Joglar J.A., Writing Committee Members (2020). Guideline for the Diagnosis and Treatment of Patients with Hypertrophic Cardiomyopathy. Circulation.

[6] American Heart Association HCM SCD Calculator. https://professional.heart.org/en/guidelines-and-statements/hcm-risk-calculator.

[7] Freitas P., Ferreira A.M., Arteaga-Fernández E., de Oliveira Antunes M., Mesquita J., Abecasis J., Marques H., Saraiva C., Matos D.N., Rodrigues R. (2019). The amount of late gadolinium enhancement outperforms current guideline-recommended criteria in the identification of patients with hypertrophic cardiomyopathy at risk of sudden cardiac death. J. Cardiovasc. Magn. Reson..

[8] Siontis K.C., Ommen S.R., Geske J.B. (2023). Art and science of risk stratification of sudden cardiac death in hypertrophic cardiomyopathy: Current state, unknowns, and future directions. Prog. Cardiovasc. Dis..

[9] Flett A.S., Hayward M.P., Ashworth M.T., Hansen M.S., Taylor A.M., Elliott P.M., McGregor C., Moon J.C. (2010). Equilibrium contrast cardiovascular magnetic resonance for the measurement of diffuse myocardial fibrosis: Preliminary validation in humans. Circulation.

[10] Wang K., Zhang W., Li S., Jin H., Jin Y., Wang L., Li R., Yang Y., Zheng J., Cheng J. (2022). Noncontrast T1ρ dispersion imaging is sensitive to diffuse fibrosis: A cardiovascular magnetic resonance study at 3T in hypertrophic cardiomyopathy. Magn. Reson. Imaging.

[11] Thompson E.W., Kamesh Iyer S., Solomon M.P., Li Z., Zhang Q., Piechnik S., Werys K., Swago S., Moon B.F., Rodgers Z.B. (2021). Endogenous T1ρ cardiovascular magnetic resonance in hypertrophic cardiomyopathy. J. Cardiovasc. Magn. Reson..

[12] Vullaganti S., Levine J., Raiker N., Syed A.A., Collins J.D., Carr J.C., Bonow R.O., Choudhury L. (2021). Fibrosis in Hypertrophic Cardiomyopathy Patients with and without Sarcomere Gene Mutations. Heart Lung Circ..

[13] Sunthankar S., Parra D.A., George-Durrett K., Crum K., Chew J.D., Christensen J., Raucci F.J., Xu M., Slaughter J.C., Soslow J.H. (2019). Tissue characterisation and myocardial mechanics using cardiac MRI in children with hypertrophic cardiomyopathy. Cardiol. Young.

[14] Huang L., Ran L., Zhao P., Tang D., Han R., Ai T., Xia L., Tao Q. (2019). MRI native T1 and T2 mapping of myocardial segments in hypertrophic cardiomyopathy: Tissue remodeling manifested prior to structure changes. Br. J. Radiol..

[15] Treibel T.A., Fridman Y., Bering P., Sayeed A., Maanja M., Frojdh F., Niklasson L., Olausson E., Wong T.C., Kellman P. (2020). Extracellular Volume Associates with Outcomes More Strongly Than Native or Post-Contrast Myocardial T1. JACC Cardiovasc. Imaging.

[16] Li Y., Liu X., Yang F., Wang J., Xu Y., Fang T., Pu L., Zhou X., Han Y., Chen Y. (2021). Prognostic value of myocardial extracellular volume fraction evaluation based on cardiac magnetic resonance T1 mapping with T1 long and short in hypertrophic cardiomyopathy. Eur. Radiol..

[17] Xu J., Zhuang B., Sirajuddin A., Li S., Huang J., Yin G., Song L., Jiang Y., Zhao S., Lu M. (2020). MRI T1 Mapping in Hypertrophic Cardiomyopathy: Evaluation in Patients without Late Gadolinium Enhancement and Hemodynamic Obstruction. Radiology.

[18] Raiker N., Vullaganti S., Collins J.D., Allen B.D., Choudhury L. (2020). Myocardial tissue characterization by gadolinium-enhanced cardiac magnetic resonance imaging for risk stratification of adverse events in hypertrophic cardiomyopathy. Int. J. Cardiovasc. Imaging.

[19] Kim P.K., Hong Y.J., Im D.J., Suh Y.J., Park C.H., Kim J.Y., Chang S., Lee H.J., Hur J., Kim Y.J. (2017). Myocardial T1 and T2 Mapping: Techniques and Clinical Applications. Korean J. Radiol..

[20] Chen S., Huang L., Zhang Q., Wang J., Chen Y. (2020). T2-weighted cardiac magnetic resonance image and myocardial biomarker in hypertrophic cardiomyopathy. Medicine.

[21] Cramer G.E., Gommans D.F., Dieker H.J., Michels M., Verheugt F., de Boer M.J., Bakker J., Fouraux M.A., Timmermans J., Kofflard M. (2020). Exercise and myocardial injury in hypertrophic cardiomyopathy. Heart.

[22] Maron B.J., Roberts W.C., Epstein S.E. Sudden death in hypertrophic cardiomyopathy: A profile of 78 patients. https://www.ahajournals.org/doi/abs/10.1161/01.CIR.65.7.1388.

[23] Liao Y.W., Redfern J., Somauroo J.D., Cooper R.M. (2020). Hypertrophic Cardiomyopathy and Exercise Restrictions: Time to Let the Shackles Off?. Br. J. Cardiol..

[24] Xu J., Yang W., Zhao S., Lu M. (2022). State-of-the-art myocardial strain by CMR feature tracking: Clinical applications and future perspectives. Eur. Radiol..

[25] Song Y., Bi X., Chen L., Yang K., Chen X., Dong Z., Wang J., Kong X., Zhao K., Wang H. (2022). Reduced myocardial septal function assessed by cardiac magnetic resonance feature tracking in patients with hypertrophic obstructive cardiomyopathy: Associated with histological myocardial fibrosis and ventricular arrhythmias. Eur. Heart J. Cardiovasc. Imaging.

[26] Cavus E., Muellerleile K., Schellert S., Schneider J., Tahir E., Chevalier C., Jahnke C., Radunski U.K., Adam G., Kirchhof P. (2021). CMR feature tracking strain patterns and their association with circulating cardiac biomarkers in patients with hypertrophic cardiomyopathy. Clin. Res. Cardiol..

[27] Zhou D., Yang W., Yang Y., Yin G., Li S., Zhuang B., Xu J., He J., Wu W., Jiang Y. (2022). Left atrial dysfunction may precede left atrial enlargement and abnormal left ventricular longitudinal function: A cardiac MR feature tracking study. BMC Cardiovasc. Disord..

[28] Liu J., Wang D., Ruan J., Wu G., Xu L., Jiang W., Wang J., Sun X., Kang L., Song L. (2022). Identification of heart failure with preserved ejection fraction helps risk stratification for hypertrophic cardiomyopathy. BMC Med..

[29] Shi R., Shi K., Huang S., Li X., Xia C.C., Li Y., He S., Li Z.L., He Y., Guo Y.K. (2022). Association between Heart Failure with Preserved Left Ventricular Ejection Fraction and Impaired Left Atrial Phasic Function in Hypertrophic Cardiomyopathy: Evaluation by Cardiac MRI Feature Tracking. J. Magn. Reson. Imaging.

[30] Yang F., Wang L., Wang J., Pu L., Xu Y., Li W., Wan K., Yang D., Sun J., Han Y. (2021). Prognostic value of fast semi-automated left atrial long-axis strain analysis in hypertrophic cardiomyopathy. J. Cardiovasc. Magn. Reson..

[31] Barbosa A.R., Dias Ferreira N., Martins O’Neill C., Ruivo C., Cruz I., Rocha Lopes L. (2020). Impaired myocardial deformation assessed by cardiac magnetic resonance is associated with increased arrhythmic risk in hypertrophic cardiomyopathy. Rev. Esp. Cardiol..

[32] Mahmod M., Raman B., Chan K., Sivalokanathan S., Smillie R.W., Abd Samat A.H., Ariga R., Dass S., Ormondroyd E., Watkins H. (2022). Right ventricular function declines prior to left ventricular ejection fraction in hypertrophic cardiomyopathy. J. Cardiovasc. Magn. Reson..

[33] Casanova J.D., Carrillo J.G., Jiménez J.M., Muñoz J.C., Esparza C.M., Alvárez M.S., Escribá R., Milla E.B., de la Pompa J.L., Raya Á. (2020). Trabeculated Myocardium in Hypertrophic Cardiomyopathy: Clinical Consequences. J. Clin. Med..

[34] Maron M.S., Rowin E.J., Lin D., Appelbaum E., Chan R.H., Gibson C.M., Lesser J.R., Lindberg J., Haas T.S., Udelson J.E. (2012). Prevalence and Clinical Profile of Myocardial Crypts in Hypertrophic Cardiomyopathy. Circ. Cardiovasc. Imaging.

[35] Wang J., Li Y., Yang F., Bravo L., Wan K., Xu Y., Cheng W., Sun J., Zhu Y., Zhu T. (2021). Fractal Analysis: Prognostic Value of Left Ventricular Trabecular Complexity Cardiovascular MRI in Participants with Hypertrophic Cardiomyopathy. Radiology.

[36] Nakamori S., Nezafat M., Ngo L.H., Manning W.J., Nezafat R. (2018). Left Atrial Epicardial Fat Volume Is Associated with Atrial Fibrillation: A Prospective Cardiovascular Magnetic Resonance 3D Dixon Study. J. Am. Heart Assoc..

[37] Zhou Y., Yu M., Cui J., Hu F., Yang Z., Yuan J., Qiao S. (2021). The predictive value of epicardial adipose tissue volume assessed by cardiac magnetic resonance for atrial fibrillation in patients with hypertrophic obstructive cardiomyopathy. Int. J. Cardiovasc. Imaging.

[38] Antiochos P., Ge Y., Heydari B., Steel K., Bingham S., Abdullah S.M., Mikolich J.R., Arai A.E., Bandettini W.P., Patel A.R. (2022). Prognostic Value of Stress Cardiac Magnetic Resonance in Patients with Known Coronary Artery Disease. JACC Cardiovasc. Imaging.

[39] Kim E.K., Lee S.C., Chang S.A., Jang S.Y., Kim S.M., Park S.J., Choi J.O., Park S.W., Jeon E.S., Choe Y.H. (2020). Prevalence and clinical significance of cardiovascular magnetic resonance adenosine stress-induced myocardial perfusion defect in hypertrophic cardiomyopathy. J. Cardiovasc. Magn. Reson..

[40] Haaf P., Garg P., Messroghli D.R., Broadbent D.A., Greenwood J.P., Plein S. (2017). Cardiac T1 Mapping and Extracellular Volume (ECV) in clinical practice: A comprehensive review. J. Cardiovasc. Magn. Reson..

[41] Li S., Zhou D., Sirajuddin A., He J., Xu J., Zhuang B., Huang J., Yin G., Fan X., Wu W. (2022). T1 Mapping and Extracellular Volume Fraction in Dilated Cardiomyopathy: A Prognosis Study. J. Am. Coll. Cardiol. Imaging.

[42] Maron M.S. (2012). Clinical utility of cardiovascular magnetic resonance in hypertrophic cardiomyopathy. J. Cardiovasc. Magn. Reson..

[43] Sugimoto T., Dulgheru R., Bernard A., Ilardi F., Contu L., Addetia K., Caballero L., Akhaladze N., Athanassopoulos G.D., Barone D. (2017). Echocardiographic reference ranges for normal left ventricular 2D strain: Results from the EACVI NORRE study. Eur. Heart J. Cardiovasc. Imaging.

[44] Kranzusch R., Aus Dem Siepen F., Wiesemann S., Zange L., Jeuthe S., Ferreira da Silva T., Kuehne T., Pieske B., Tillmanns C., Friedrich M.G. (2020). Z-score mapping for standardized analysis and reporting of cardiovascular magnetic resonance modified Look-Locker inversion recovery (MOLLI) T1 data: Normal behavior and validation in patients with amyloidosis. J. Cardiovasc. Magn. Reson..

